# SWISS CENTENARIANS: PRELIMINARY FINDINGS FROM THE SWISS100 PHONE STUDY

**DOI:** 10.1093/geroni/igac059.2166

**Published:** 2022-12-20

**Authors:** Daniela Jopp, Stefano Cavalli, Armin von Gunten, Francois Herrmann, Mike Martin, Kim Uittenhove

**Affiliations:** University of Lausanne, Lausanne, Vaud, Switzerland; University of Applied Sciences and Arts of Southern Switzerland, Manno, Ticino, Switzerland; Lausanne University Hospital, Prilly, Vaud, Switzerland; Geneva University Hospitals, Thônex, Geneve, Switzerland; University of Zurich, Zurich, Zurich, Switzerland; University of Lausanne, Lausanne, Vaud, Switzerland

## Abstract

Increase in very old individuals is observed in all developed countries around the world. The number of centenarians has also been rising, requiring the investigation of the characteristics of these exceptionally long-lived individuals as well as their experience of life at age 100. In the present study, we present findings from the first nation-wide Swiss centenarian study SWISS100. Given the ongoing COVID-19 pandemic, we conducted a telephone study with centenarians and a family member as proxy informant, using a mixed-methods approach to investigate specific characteristics, their life circumstances and their experience during the pandemic. Recruitment was conducted with the help of the national address registry. A total of 64 centenarians and 62 family members participated, leading to data for 119 centenarians. Centenarians were on average 102 years old, with a range of 100 to 108 years. In line with higher survival rates in females, 76% were women and 24% were men. Most centenarians had received basic education and had completed an apprenticeship. Concerning their residence, 43% lived in private homes and 57% lived in institutions. Of those living in private, half lived alone, one fourth lived with their spouse and one fourth lived with a child. The majority was widowed. Over 80% had children. Although over 70% of the centenarians reported health restrictions, 60% reported good to excellent subjective health. Over 90% of the sample were aware of COVID-19. Despite substantial COVID-19-related restrictions, life-satisfaction was high. Overall, Swiss centenarians show health-related vulnerability but also psychological resilience.

